# Opening Lecture, Baltimore College of Dental Surgery, Session 1904-5

**Published:** 1904-11

**Authors:** B. Holly Smith


THE AMERICAN

JOURNAL OF DENTAL SCIENCE

### OPENING LECTURE, BALTIMORE COLLEGE OF DENTAL SURGERY, SESSION 1904-5.

B. HOLLY SMITH, M. D., D. D. S.

Gentlemen:—I bring to you the cordial greetings of the faculty. We welcome you of the senior and junior classes as old friends. Some of you have journeyed far in your vacations. All, we hope, are here with renewed strength and spirit for the work of another college year. How fortunate it is that even when the task is difficult, a little rest and change bring us to its undertaking with an earnest determination for its accomplishment. Then, too, it should be pleasant to feel a comradeship and sympathy in the work; we are here to undertake it together,—teachers, students, all for it and at it with a will.

To the freshmen I bring a most friendly welcome. You, as strangers, we especially wish to make feel at home, to introduce you to the work in such a way that it may seem attractive; to encourage your every hope and ambition for earnest and fruitful work. Some of the training will no doubt be strange and confusing. Be patient and don't expect to do too much at once, though lose no time in taking hold of any threads within your grasp.

As it is the custom of those in authority on fete days and festival occasions to give gifts and announce promotions, I present from the faculty to the sophomore class promotion to the junior class, and that there may be no invidious distinction, the juniors are promoted to the senior class and may hope for graduation this year. Now this is a wonderful exhibition of generosity on the part of the faculty, and it is hoped that it will be appreciated, as it is the first time in the history of the school that such wholesale advancement has been granted.

You are all here to make the best of your opportunities in the study of Dentistry, and most, we must assume, look upon the effort as something to bring occupation, livelihood and position in the world. The accidents which influence the selection of a vocation are too many for us to assume that you all come charged with an adequate knowledge of what the profession of Dentistry really is. If you have rightly chosen your profession, however, it cannot fail to be of interest to you to glance back at its history and at those changes that have taken place in the methods and in the preparation for it as a calling.

With the time allotted me tonight it is manifestly impossible to go into its ancient history. The ingenuity and resourcefulness of man in all ages has been exhibited, in some effort to assist nature mechanically, that is, to furnish some artificial substitute for the losses resulting from disease and accident; but as far as recorded, these efforts have been desultory and not based on any scientific record or mechanical training, since neither tradition, records nor mummified or fossilized remains of early ages, show any similarity or concerted procedure.

Hippocrates, about four hundred years before the Christian era, is about the first whose writings show any appreciation or recognition of diseases of the mouth and teeth, and their relation to diseases in general. In 1716 we have a record of a dentist, Laudemiey of Paris, being called upon to perform an operation on the mouth of Philip V of Spain, and in 1723 he announced himself dental surgeon to his catholic majesty, King Philip V of Spain. In 1728 Fouchard published two volumes denominated "The First Attempt to Systematize and Treat Methodically the Dental Art." Many others attained eminence in France, among them Jourdain was so highly esteemed by the medical men that they frequently had him in consultation. Later in England, Hunter published a treatise on dentistry, to be followed afterwards by Fox and Blake.

During the Revolutionary War there came to this country one Le Maire, a Frenchman, who was the first among us to show superior skill in dental art; he not only did some acceptable work but gave training to others. Among other importations we had Gardette from France, Wolfingdale from England, Edward Hudson of Dublin. Mr. Greenwood was about this time perhaps the most eminent native dentist, conspicuous for his distinguished service to George Washington.

These individual practitioners, standing aloof from each other, often bitterly jealous and unfriendly, gave little promise of any help in the development of professional dentistry.

There are three significant factors in the growth of what we now recognize as professional dentistry: first, the organization of this, the first dental college; second, the publication of the first dental journal; third, the organization of the first dental society. The schools, of course, were essential to train men not only as specialists on the diseases of the mouth and jaws, but to give them that skill in the use of tools and materials utterly unknown to the general practitioner of medicine; the dental journal to publish to the world the researches and accomplishments of the profession, as well as to acquaint each member with what was being done by the others; while the dental society brought the profession in touch one with another, and beside an improvement in materials and methods, made possible that esprit de corps, or professional ethics which has lifted us above the sordid methods of the commercial classes. These have all contributed to make dentistry what it is today, and lacking any of them its growth would have been less rapid.

Before the inception of these enterprises, dentistry was a cult or calling with many able practitioners, it is true, but practically without exponents. Demonstrations were rare, methods of practice were secret; materials and their individual manipulation were guarded carefully from all rivals. The dental laboratory was a locked room and could only be entered by the apprentice after an ironclad oath never to reveal its secrets. Indeed, surgery itself was discredited, and in many countries, "saw-bones" as much a term of reproach as "tooth-carpenter."

Strange to say the dental college, journal and semi-national society were in great part, the work of two men who were at that time Baltimoreans,—Chapin A. Harris and Horace M. Hayden. These men were hard workers, great students and soon to be prolific dental authors and translators of works on medicine and kindred subjects. Harris was a man of powerful physique, great pertinacity of purpose, and I have often been told by those associated with him, he had greater capacity for work than any man they had ever known. So it seems to have been with the pioneers of all ages, the task is set, the courage and capacity given. Harris had great personal dignity associated with no little vanity and self-confidence. He recognized his own qualities as a leader and was not a little jealous of his reputation as such. His extreme sensitiveness to criticism involved him in many contentions, some of which assumed great bitterness.

Hayden was a much older man. The fires of his youth had burned out in the many enterprises of his early life. He had been a sailor, traveler, geologist, mineralogist, dentist, doctor of medicine and prolific author.

An item in the book review of the *New York Times* of recent date, states that as much as $500 has lately been offered for a single treatise of his on geology. He was unquestionably a man of broader culture and greater learning than Harris, but his advanced years and more retiring disposition had to succumb to the virile domination of the latter, and in the early history of their association, Harris was the master.

The medical associates with these men, Bond and Baxley, were experienced lecturers and authors; men of force and power in their profession and community.

It is not strange then, so well manned was this new faculty, that support and endorsement came from many quarters. There was little money, however, and poor equipment. Part of the work of the department of anatomy was conducted in a stable-loft. For the first year, I have been told, the instruction was "mostly wind," very few practical demonstrations or clinical operations were performed. This was corrected soon, however, and from those days to these,—with perhaps the exception of the period during the Civil War,—the history of the B. C. D. S. has been one of uninterrupted effort and successful achievement.

It is interesting to note the various changes that have taken place in the conditions upon which the dental diploma has been awarded. The first sessions of the early dental schools were of about four months' duration. For the beginners, two of these short sessions were demanded, but there were many men in dental practice whose proficiency was recognized and either an honorary degree granted, or they were graduated after an examination. The sessions were subsequently extended to five months and a credit of one term granted to those who had been in practice five years. This was often abused and by a stretch of imagination, collecting dental bills, doing chores about a dental office, or sitting about watching a vulcanizer, was taken in lieu of a genuine practice of dentistry.

This condition prevailed until the late dean of this school, Dr. R. B. Winder, a man of superior judgment and ability, conceived the necessity for uniting all the colleges into one organization, so that legislation might be enacted, raising the standard of dental teaching and requirements. He called a conference of deans of Philadelphia and New York schools, and out of this developed the National Association of Dental Faculties, which association has since controlled the standards of entrance and teaching requirements. The five years' practice as equivalent to one session at college, was quickly abolished; the bestowal of honorary degrees met the same fate, and six months' session substituted for five. Later on the session was lengthened to seven months, and a three year course took the place of the two year, until finally in the fervor for extending the course, four years was named. It was found, however, that so many considered this impractical and unnecessary, that the Association has rescinded this requirement and returned to three years of seven months.

Preliminary requirements were unknown in the early days of the profession, but have persistently increased of late until by the next session, or perhaps the one following, students will be compelled to have a high school education, at least, before beginning the study of dentistry.

The methods of teaching have greatly improved. Technical instruction and demonstration increased, subjects added to the curriculum. Thus rapidly dental training has increased in efficiency; now what must be the effect upon the product, the dental graduate? He will not find himself in competition with unprepared men, and he will not succeed if he himself is unprepared.

*The American Journal of Dental Science* was the first dental journal published, and was the outgrowth of a plan to bring the honorable practitioners of dentistry together, liberalize their views of practice, as well as afford a means of publishing the proceedings of the Dental Society and the lectures and addresses of the professors of the B. C. D. S.

Its publication was begun in 1839 and was accomplished by a board of editors,—Eleazar Parmly, Elisha Baker and Solyman Brown. Subsequently Chapin A. Harris and Solyman Brown were its editors. The first volume was published by the private enterprise of these men; afterward, until 1850, the journal was published under the auspices of the American Society of Dental Surgeons, and the subscription price raised from $3.00 to $5.00.

Many books on dental subjects which had been published before, were here republished, necessary translations being made by professors of the B. C. D. S. Contributions were invited and published from dentists at home and abroad. Its pages are interesting reading yet, and evidence literary style and a learning that will compare favorably with any publication of its day. This journal has had almost a continuous publication until the present time, though it has changed hands several times.

Many other journals were added to this one, such as "The News Letter" (now out of publication), "Dental Cosmos," "Dental Digest," "Dental Review," "Dental Luminary," "International Dental Journal" and numerous others.

These journals, most of them exceptionally well published and edited, are, with the exception of the International Dental Journal,* published by supply houses. This is a source of great regret and mortification to many practitioners of dentistry. They believe the journals should be in the hands of the dental societies, and consequently entirely free from any connection with dental dealers. The difficulty has always been the expense. Illustrations are expensive. The "International" has had a hard fight, as there are so many competitors in the field, and is not yet free from debt. It cannot be denied that this association with commercialism has been more or less profitably employed in most dental publications. So tactful, so liberal, so yielding, has the management been, that objections have grown to be regarded as mere sentiment. No scientific journals have given greater evidence of merit (from the publishers' standpoint), illustrations have been abundant, editing capable; and yet in some quarters there is a feeling that it would be more dignified to have the profession represented by some such independent publications as the "American Medical Association," or "American Medicine."

It seems to me fit opportunity to suggest to you young men that this is an auspicious moment to start some such organ here in our midst, the "Journal of the B. C. D. S. and Its Alumni." You might start it as a quarterly. The bright things you might write would be gladly received by the graduates, as well as matriculates of the school. Then, too, you would serve to establish a system of communication between your fellows of long ago and your alma mater.

As early as 1817, Dr. Hayden proposed the formation of a national dental society, and visits were made to Philadelphia, New York and Boston to further this purpose, and yet it was not until 1840 that some fifteen or twenty men met in New York to organize the "American Society of Dental Surgeons." Dr. Hayden was elected the first president; Dr. Harris, secretary.

Three years later, this association numbering one hundred and twenty-five members, met in Baltimore, and Dr. Harris in his address said, "In all great movements, either in religion, politics, science or benevolence, the American character and the popular genius of all our institutions immediately direct us to lay hold of the great principle of voluntary association, as an agent of stronger moral power than any known in the country, to effect any desirable good. We have no wealthy and powerful privileged orders to command any given amelioration or improvement, and hence we combine the power of popular number and bring it to bear upon the objects we desire to accomplish. This national peculiarity accounts for the numerous benevolent and scientific associations found in the United States, before the moral power of which vice, ignorance, empiricism, bigotry, oppression, and long-established habits and modes of thinking are fleeing away like mists of morning be-

---

*The American Journal of Dental Science already referred to in this paper, is also an independent dental magazine, being owned, edited and published by members of the dental profession.—Editor.

fore the rising sun. This will furnish the reason why America can boast of the first society of Dental Surgeons ever associated together in the world; and of this association shall produce results in any manner comparable to those produced by other American associations, now the wonder and praise of the world, the character of an American dental surgeon will stand as high on the archives of science as do the names of Washington, Franklin, Hancock, Henry and Jefferson on the roll of freedom, or Mills, Judson, Perkins and Hill in the annals of American missionary benevolence."

Now, while the oratory of the paragraph just quoted may be a little too fervid to suit the modern idea of reserve in public speech, yet it may serve to bring back to us the florid style of a by-gone time, and give us some idea of the reverence in which the man held his calling, and the energy he put into his work for its uplift.

The society before which he made this address demanded unqualified evidence of moral character as well as practical ability. Its fees were of two kinds: ten dollars for members of acknowledged standing and ability in the profession and twenty-five dollars for beginners who underwent an examination preliminary to their admission. It had a continuous and useful life of sixteen years, when it was finally dissolved through bitterness engendered by contentions about the use of amalgam. Many prominent members held that the use of this now indispensable material was equivalent to malpractice.

The American Society of Dental Surgeons was succeeded by the American Dental Convention, a body without constitution or by-laws, a simple, open meeting or conference.

This organization was of short duration, and was itself succeeded by the American Dental Association, which held its first meeting at Niagara Falls, 1859. This was from the first a delegate body and had a successful and continuous existence until merged with the Southern Dental Association, into what we now proudly recognize as the National Association.

For over twenty years it had been considered desirable by many to have all sections of the country involved in one central organization. The Southern Dental Association had grown into almost equal importance with the American. Effort after effort had been made to unite the two, but the most that had been accomplished was to confer. Antagonisms partly engendered by the Civil War might be held responsible, though many Southern men were members of the American.

Finally I, as president of the Southern Dental Association, at a meeting held at Old Point Comfort, recommended in the annual address that committees of the two associations be appointed to consider this most desirable object. Dr. Thomas Fillebrown of Boston, a prominent member of the American and others were enthusiastic in support of the idea. Thanks to the ripeness of the time and more thanks to the persistent and untiring efforts of the many loyal advocates of this cause, the union of the two associations was accomplished. Dr. Fillebrown has never received half the credit he is entitled to. Both committees worked hard on matters of detail, but his efforts were unceasing. With the hearty co-operation of all of us who were friendly to this cause, success was finally achieved and the National Dental Association held its first meeting at Old Point Comfort in 1895, with Dr. Fillebrown as its first president.

The consummation of these efforts secured to America for the first time one authoritative body whose decisions upon any scientific subject, matter of practice, or question of policy, are accepted the world over; as witness the question of the formation of the Fourth International Congress. The National Association appointed a committee of organization for the Congress. The Federation Dentaire Internationale, an association with seventeen nations represented in membership, also appointed a committee. Much dispute and contention arose as to authority, but it was decided that the National Association through its committee should organize the Congress, and it resulted in the largest and most successful congress in the history of dentistry.

We have not time here to take up the history of any of the many prominent local societies, but they are doing good work; and right here I will say that when you are graduated, your loyalty to your profession and your fidelity to the teachings of your alma mater, demand that you lose no time in associating yourselves with one of these bodies; and your ambition should not stop short of being a delegate to the National. Your association with dental societies will prove a post-graduate course of study, which will more than repay you for any expense or sacrifice you make; and we, as your teachers, will feel proud that you have not fallen into that selfish oblivion which has engulfed so many, and which shows such small appreciation of your advantages.

Our parental relation will not allow us to close this address without a personal word directed especially to the stranger who is within our gates; but to you all I bring the admonitions which you may find appropriate.

This is the beginning of a life that is to be different from the old life at home. It should be to all of you a pleasant and happy prelude to the mature years of responsibility and professional labor which await you. I counsel you to make the most of your opportunities in your college life. First, fritter not the days away in idleness, guard well your conduct toward your fellow, lose no chance to make and keep a friend. Be not unmindful of what is expected of you from home. Do not neglect any opportunity which the school offers for your advancement in your studies. Live sane and wholesome lives. Do these things, and the memory of your college days will bless you throughout the remainder of your life.

This is a happy time to begin the study of dentistry. At no time in the history of the world has there existed such a wide field of usefulness for the dentists nor such a keen appreciation awaited his successful practice. Only a short time ago the dentist, in common with the general surgeon, had little of the respect and honor freely accorded the general practitioner of medicine. Today these distinctions are forgotten in a general appreciation of his helpfulness and culture. When your labors shall happily culminate in the bestowal upon you by our worthy dean of a scroll bearing your name and ours, you will hear him say that he gives it "with all its rights and privileges and subject to all its responsibilities." If then this is a propitious time to begin to prepare for the practice of dentistry, it is also a time when more, much more, is expected of you. To guard against any unworthy entrance to your profession, more time is spent in your training, rigid examinations to test your fitness are given by your college, and finally after you have graduated, a Dental Examining Board will have to pass upon your fitness before you will be allowed to practice.

To prepare for these tests, the beginning must be made now, not next month or next week, but tonight the resolution must be made. There is no royal road to success in this nor any other department in life, but may be attained only by what Dr. Osler calls the "secret of life as I have seen the game played." Some years ago he said to a body of students,—"I propose to give you the master word of success. Though a little one, it looms large in meaning. It is the open sesame to every portal, the great equalizer in the world, the true philosopher's stone which transmutes all the base metal of humanity into gold. The stupid man among you it will make bright, the bright man brilliant and the brilliant man steady. With the magic word in your heart all things are possible, and without it all study is vanity and vexation. To the youth it brings hope, to the middle-aged confidence, to the aged repose. It is directly responsible for all the advances in medicine during the past 25 centuries. * * * Not only has it been the touchstone of progress, but it is the measure of success in every-day life.

"And the master-word is WORK—a little one, as I have said, but fraught with momentous sequences if you can but write it upon the tables of your heart and bind it upon your forehead."

